# The paradox of hikikomori through a transcultural lens

**DOI:** 10.1192/bji.2024.38

**Published:** 2025-02

**Authors:** Yoko Nagai, Amy Kartar, Magdalena Pfaff, Hussien Elkholy

**Affiliations:** Clinical Neuroscience Department, Brighton and Sussex Medical School, University of Sussex, Brighton, UK

**Keywords:** Hikikomori, social withdrawal, transcultural, COVID-19, paradox

## Abstract

This article appraises cultural understanding and controversies regarding hikikomori (prolonged social withdrawal), with reference to research over the past 20 years. Initially viewed as a uniquely Japanese phenomenon, hikikomori is now recognised globally, prompting a re-evaluation of its cultural, psychological and socioeconomic demographics. A revision in lifestyle after the COVID-19 pandemic and ongoing technological advancements – particularly the rise of social media, gaming and the internet – have paradoxically both exacerbated isolation and provided new forms of social interaction for young adults who confine themselves at home. This phenomenon underlines the complex interplay between putative individual psychopathology, neurodiversity and broader societal shifts across the globe.

In 1998, the Japanese psychiatrist Tamaki Saito coined the term hikikomori to describe a specific severe form of social withdrawal that had been increasingly observed in Japan. Saito initially defined hikikomori as staying at home for 6 months or more and avoiding participating in social activities, for reasons unexplained by other mental disorders. Until recently, hikikomori was viewed as a culture-bound syndrome related to the Japanese culture of overprotection of children and acceptance of overdependent behaviour.^1^ The social withdrawal can be so severe that family members prepare meals for the individual and leave them outside their door, as portrayed in Japanese films and media. This level of isolation might be difficult to understand in different cultural contexts. However, social withdrawal is a phenomenon that has been described in the context of several psychiatric disorders. Although the Japanese Cabinet Office had estimated that around 1.46 million people had hikikomori, a prevalence rate of approximately 1%, a regional study in 2020–2021 identified an actual prevalence rate of 2.3% in 15- to 64-year-olds.^2^

## The evolution of understanding of hikikomori

In the mid-2000s, one of us (Y.N.) presented an introduction to the concept of internet-based psychotherapy to the Japanese parliament as a potential accessible intervention for what appeared to be an epidemic of hikikomori. Almost 20 years later, hikikomori remains a significant problem and it is now recognised and studied as a global phenomenon rather than a culturally specific condition.^2–4^

The definition of hikikomori has evolved from Saito's original description, in consideration of the severity of the confinement, acknowledging that some individuals may leave their homes despite avoiding social interactions.^5^ The distinction is also made between the pathological and non-pathological (no significant functional impairment and distress associated with the social isolation) expression of hikikomori.^1^ Moreover, although the initial Japanese Cabinet Office's definition of hikikomori excluded individuals with physical and mental disorders, some researchers have emphasised the importance of considering co-occurring conditions.^6–8^ It is argued that hikikomori, as a behaviour, tends to co-occur with other diagnoses, and therefore comorbidities should not be used as exclusion criteria.^4^ Perhaps the most important/critical relationship is with neurodevelopmental disorders, particularly autism.^9^ Thus, the concept of ‘secondary hikikomori’ was proposed to define such cases, where severe social withdrawal behaviour is a manifestation or consequence of another psychiatric disorder.^10^ However, for some conditions, it could be argued that it would be difficult to identify whether hikikomori is indeed secondary or whether it is the primary disorder. Consequently, a standardised definition is important for both clinical and research perspectives.^4^ One approach might be to view the expression of hikikomori as a spectrum, where the variation in severity of the condition can be manifested by level of confinement and impact on function. This approach allows identification of comorbid conditions, while appreciating that symptoms are likely to overlap and may inform about their potential effect on expression of hikikomori. Interestingly, there is also a proposal to consider hikikomori as a diagnostic specifier rather than a stand-alone condition, so that certain mental health conditions can present with or without hikikomori-like social isolation.^11^

## Transcultural view of hikikomori

There is now a consensus that the general hikikomori phenomenon is not confined to Japan; it also exists across the globe.^2–4^ This raises questions about the relevance of traditional Japanese culture as a causal link to hikikomori.

Family structure and relationships between parents and their children vary across cultures. ‘Amae’ refers to an overdependent and enmeshed parent–child relationship and is an extensively studied aspect of Japanese culture that was proposed to play a role in the development of hikikomori.^4^ Interestingly, the same concept may apply in Middle Eastern cultures, where extended family structure and similar acceptance of dependency are noted,^12^ although to our knowledge only one case report from Oman^3^ has been published to date. However, it is pointed out that maladaptive and dysfunctional family behaviour regardless of cultures (the absence of effective communication and emotional interaction, coupled with a lack of empathy towards children) contributes to the formation of hikikomori.^13^

Hikikomori is also suggested to be linked to excessive video/computer gaming, but this relationship is complex.^1^ It is currently not clear whether a causal link between hikikomori and pathological computer gaming exists, and it is likely to present a chicken and egg conundrum. While some researchers consider hikikomori to be a risk factor for pathological gaming, others argued that the use of games during early hikikomori could be a coping strategy by having some social involvement in the virtual world.^1^ However, it remains uncertain whether the current worldwide increases in gaming will lead to a prevalence of hikikomori in other countries that is comparable to that in Japan. This could be because hikikomori is related to other factors, including cultural ones.

Writing this article from within the UK, it should be noted that hikikomori has increasing relevance, as is likely in many other Western countries. This is particularly shaped by social and technological changes and economic crises. The coronavirus-19 (COVID-19) pandemic led to severe restrictions and social isolation, which might have contributed to the emergence of hikikomori-like behaviour in young adults. Early adulthood remains a critical window for possible onset of hikikomori,^1,2^ and young people were disproportionately disadvantaged by COVID-19 restrictions.^14^ Now that the pandemic is largely over and individuals are re-engaging with the world, it is likely that we will witness the emergence of hikikomori or increased case numbers. It is thus of critical importance that hikikomori is recognised by Western psychiatry, as the number of cases is likely to have increased in line with related diagnoses, such as autism that features pathological demand avoidance, which have been increasingly identified over the past 20 years in the UK.^15^ Furthermore, more than a decade ago a high incidence of internet addiction and compulsive internet use (63%) was found in an adult British sample.^16^ These disorders are recognised comorbidities with hikikomori, which poses the question whether hikikomori numbers are unknowingly growing. Research into hikikomori is still limited in the UK and the term itself is not widely recognised among Western psychiatrists. Even though social withdrawal appears to be a cardinal presentation of psychological distress among young adults in Japan, it may not be the primary manifestation in the other countries, where different mental health presentations may be more evident.^17^

## Future perspectives

Over the past 20 years, global awareness of hikikomori has grown significantly, reflecting an increasing number of young individuals of working age withdrawing from society. The normalisation of staying at home and its perceived acceptability during the COVID-19 pandemic is likely to have further encouraged this pattern of coping behaviour, with social media use serving as a compensatory mechanism for mental health problems.^18^ Since ‘hikikomori’ is not yet a recognised diagnostic term in DSM-5 or ICD-11, extreme cases of withdrawal outside of Japan are likely to be classified among other mental health conditions, including depression, anxiety disorders or agoraphobia. Although the globalisation of the concept of hikikomori highlights concerns about the mental health of young people, the causation of social withdrawal and its presentation and treatment options are still likely to reflect cultural differences. A key global recognition is that the rise in hikikomori cases, combined with a declining birth rate in affected countries, will significantly influence social demographics. The resulting decrease in the workforce will have a profound impact on future social care systems. In Western societies, including the UK, a systematic investigation of the prevalence of severe social withdrawal of young adults is necessary, and increasing the awareness of hikikomori and recognition of its risk factors in different societal and cultural contexts will aid understanding and available treatment options for many people currently distant from care.

## Data Availability

Data availability is not applicable to this article as no new data were created or analysed in this study.
